# The role of serine protease HtrA in acute ulcerative enterocolitis and extra-intestinal immune responses during *Campylobacter jejuni* infection of gnotobiotic IL-10 deficient mice

**DOI:** 10.3389/fcimb.2014.00077

**Published:** 2014-06-10

**Authors:** Markus M. Heimesaat, Marie Alutis, Ursula Grundmann, André Fischer, Nicole Tegtmeyer, Manja Böhm, Anja A. Kühl, Ulf B. Göbel, Steffen Backert, Stefan Bereswill

**Affiliations:** ^1^Department of Microbiology and Hygiene, Charité - University Medicine BerlinBerlin, Germany; ^2^Division of Microbiology, Department of Biology, Friedrich Alexander University Erlangen/NurembergErlangen, Germany; ^3^Department of Medicine I for Gastroenterology, Infectious Disease and Rheumatology/Research Center ImmunoSciences, Charité - University Medicine BerlinBerlin, Germany

**Keywords:** ulcerative enterocolitis, colonization resistance, innate immunity, host-pathogen-interaction, bacterial translocation, intestinal immunopathology, extra-intestinal immune responses, systemic immune responses

## Abstract

*Campylobacter jejuni* infections have a high prevalence worldwide and represent a significant socioeconomic burden. *C. jejuni* can cross the intestinal epithelial barrier as visualized in biopsies derived from human patients and animal models, however, the underlying molecular mechanisms and associated immunopathology are still not well understood. We have recently shown that the secreted serine protease HtrA (high temperature requirement A) plays a key role in *C. jejuni* cellular invasion and transmigration across polarized epithelial cells *in vitro*. In the present *in vivo* study we investigated the role of HtrA during *C. jejuni* infection of mice. We used the gnotobiotic IL-10^−/−^ mouse model to study campylobacteriosis following peroral infection with the *C. jejuni* wild-type (WT) strain NCTC11168 and the isogenic, non-polar NCTC11168Δ*htrA* deletion mutant. Six days post infection (p.i.) with either strain mice harbored comparable intestinal *C. jejuni* loads, whereas ulcerative enterocolitis was less pronounced in mice infected with the Δ*htrA* mutant strain. Moreover, Δ*htrA* mutant infected mice displayed lower apoptotic cell numbers in the large intestinal mucosa, less colonic accumulation of neutrophils, macrophages and monocytes, lower large intestinal nitric oxide, IFN-γ, and IL-6 as well as lower TNF-α and IL-6 serum concentrations as compared to WT strain infected mice at day 6 p.i. Notably, immunopathological responses were not restricted to the intestinal tract given that liver and kidneys exhibited mild histopathological changes 6 days p.i. with either *C. jejuni* strain. We also found that hepatic and renal nitric oxide levels or renal TNF-α concentrations were lower in the Δ*htrA* mutant as compared to WT strain infected mice. In conclusion, we show here that the *C. jejuni* HtrA protein plays a pivotal role in inducing host cell apoptosis and immunopathology during murine campylobacteriosis in the gut *in vivo*.

## Introduction

*Campylobacter* species are classical zoonotic pathogens, living predominantly as commensals in the gastrointestinal tract of a wide range of birds and mammals, including agriculturally important animals (Young et al., 2007; van Putten et al., 2009; Dasti et al., 2010; Gaynor and Szymanski, 2012). Thus, contaminated animal food products serve as a major source of *Campylobacter* infections in humans (Alter et al., 2011; Oyarzabal and Backert, 2011). The most prevalent *Campylobacter* species in human disease is *C. jejuni*, which represents the leading cause of bacterial infections in the gut and acute diarrheal disease worldwide (Friedman et al., 2000; Young et al., 2007; Mukhopadhya et al., 2011). Disease outcome in humans varies from mild, non-inflammatory, self-limiting diarrhea to severe, inflammatory, bloody diarrhea lasting for several weeks (Young et al., 2007; Oyarzabal and Backert, 2011). In addition, in a minority of infected persons, *C. jejuni* can be associated with the development of reactive arthritis and peripheral neuropathies, the Miller–Fisher and Guillain–Barrè syndromes, respectively (Nachamkin et al., 2008; Szymanski and Gaynor, 2012). *In vivo* and *in vitro* research studies performed in the last two decades revealed that *C. jejuni* exhibits various remarkable properties during infection. An important feature of *C. jejuni* is their ability to bind to and enter human gut epithelial cells causing intestinal tissue damage (Ó'Cróinín and Backert, 2012). *C. jejuni* adherence to epithelial cells has been shown to involve a variety of proposed and confirmed outer membrane adhesins, including JlpA, PEB1, CadF, FlpA among others (Pei et al., 1998; Konkel et al., 2005; Poly and Guerry, 2008; Novik et al., 2010; Eucker and Konkel, 2012). *C. jejuni* invasion of cultured INT-407 and other cell lines has been observed to induce rearrangements of the host cytoskeleton by small Rho GTPases, Rac1, and Cdc42, which are directly linked to bacterial uptake (Krause-Gruszczynska et al., 2007a, 2011; Boehm et al., 2011; Eucker and Konkel, 2012). *C. jejuni* can also cross the intestinal epithelial barrier as visualized in biopsies derived from human patients (Backert et al., 2013). Efforts with rodent and chicken infection model systems have been made to study pathogenicity mechanisms of *C. jejuni in vivo*, but each animal system has diverse limitations. Currently, disease manifestation such as gastroenteritis mimicking human camplylobacteriosis can be achieved in distinct infection models such as conventionally colonized infant wild-type (WT) and gnotobiotic IL-10^−/−^ mice (Gaynor and Szymanski, 2012; Haag et al., 2012a,b). Notably, when infecting with a *C. jejuni* B2 strain (which is well known for its effective colonization properties) immediately after weaning, approximately 90% of conventionally colonized 3-weeks-old infant mice developed self-limiting enterocolitis within 6–8 days resolving within 2 weeks post infection (p.i.) (Haag et al., 2012b). However, when infecting with other *C. jejuni* strains than B2 such as strains 81–176 or 11168, a huge variability in colonization and disease development could be observed in the infant mouse model. Irrespective of the *C. jejuni* strain, however, gnotobiotic IL-10^−/−^ mice get readily colonized by the pathogen at high loads following peroral infection. Very similar to immunocompromised patients, infected mice develop non-self limiting wasting ulcerative enterocolitis within 1 week p.i. (Haag et al., 2012a; Heimesaat et al., 2014). Importantly, the intestinal inflammation induced by *C. jejuni* in mice and humans is aggravated by *C. jeuni* lipooligosaccharide (LOS) via Toll-like-receptor-4 (TLR-4), as we could show previously (Haag et al., 2012a).

We and others have recently reported that the HtrA (high temperature requirement A) protein of *C. jejuni* is a novel virulence factor (Brøndsted et al., 2005; Bæk et al., 2011a,b; Boehm et al., 2012; Hoy et al., 2012). Bacterial HtrA proteins represent a class of conserved heat shock induced serine proteases with additional chaperone activity, which were shown to have a significant impact on the virulence capabilities of various bacterial pathogens (Ingmer and Brøndsted, 2009; Backert et al., 2013; Frees et al., 2013; Skorko-Glonek et al., 2013). For example, it was demonstrated that growth of the Δ*htrA* mutant was severely impaired at 44°C as compared to WT *C. jejuni* and tolerance of the mutant bacteria against oxygen stress is strongly reduced (Brøndsted et al., 2005). In many different bacterial species, HtrA proteins are localized in the periplasm, where they form proteolytically active multimers with crucial function in the intracellular protein quality control machinery (Clausen et al., 2002, 2011). The class of HtrA proteins typically consists of a signal peptide, a trypsin-like serine protease domain and one or two PDZ [post synaptic density protein (PSD95), Drosophila disc large tumor suppressor (Dlg1), and zonula occludens-1 protein (ZO-1)] domains for protein-protein interactions (Kim and Kim, 2005). For long time it was assumed that HtrA family members are strictly acting intracellularly within the bacteria. However, very recently we have discovered a remarkably new feature of HtrA during infection. In various ε-proteobacteria such as *H. pylori* and *C. jejuni*, HtrA is actively secreted into the extracellular environment, where it cleaves the cell surface adhesion protein and tumor-suppressor E-cadherin (Hoy et al., 2010, 2012; Boehm et al., 2012, 2013). Infection experiments with *C. jejuni in vitro* indicated that HtrA can open the cell-to-cell junctions in cell monolayers by cleaving-off the ~90-kDa extracellular NTF domain of E-cadherin (Boehm et al., 2012; Hoy et al., 2012). Deletion of the *htrA* gene leads to a defect in E-cadherin shedding and transmigration of *C. jejuni* across monolayers of polarized human MKN-28 epithelial cells *in vitro* (Boehm et al., 2012). However, the potential relevance of the *htrA* gene for the interaction of *C. jejuni* with the host immune system has not been investigated so far.

To address this important question, we applied in the present study the *C. jejuni* infection model system of gnotobiotic IL-10^−/−^ mice. Here we investigated the role of *C. jejuni* HtrA in (i) colonization capacity, (ii) translocation, (iii) clinical outcome, (iv) intestinal inflammation, and (v) extra-intestinal sequelae including systemic immune responses following infection of gnotobiotic IL-10^−/−^ mice with the *C. jejuni* WT strain NCTC11168 and the isogenic knockout mutant strain NCTC11168Δ*htrA*.

## Materials and methods

### Ethics statement

All animal experiments were conducted according to the European Guidelines for animal welfare (2010/63/EU) with approval of the commission for animal experiments headed by the “Landesamt für Gesundheit und Soziales” (LaGeSo, Berlin, Germany; registration numbers G0123/12). Animal welfare was monitored twice daily by assessment of clinical conditions.

### *C. jejuni* strains and genetic complementation of *Htra*

For genetic complementation of *htrA*, we used the pCam-148 chromosomal *C. jejuni* complementation vector kindly provided by Dr. Dennis Linton (University Manchester, UK). Briefly, pCam-148 contains a 2,178 bp fragment of *C. jejuni* NCTC11168 genomic DNA (position 205,297 to 207,475) cloned into the *Sma*I restriction site of plasmid pUC18. pCam-148 contains a singular *Spe*I restriction site in the *C. jejuni* sequence within the pseudogene downstream of Cj0208. We used this *Spe*I site to introduce three additional restriction sites (*Not*I, *Mlu*I, and *Nru*I) using the primer annealing approach. Subsequently, we amplified by PCR a 1,694 bp fragment of the *htrA* gene of *C. jejuni* NCTC11168, including 200-bp upstream and 75-bp downstream sequences using the primers HtrA-1 5′GTTATATTTCCTTAAAAATTTTTAC and HtrA-2 5′AGTTTTCCTTTATTTTAAACTTAAT. The resulting PCR product was cloned into the pSB-249 vector containing flanking *Not*I and *Mlu*I sites, respectively. The *htrA* gene was then further subcloned into the *Not*I and *Mlu*I sites of pCam-148. As a selection marker, we used a 795-bp kanamycin-resistant Aph cassette with its own promoter from plasmid pRYSK12 (kindly provided by Dr. Sabine Kienesberger, University Graz, Austria). This Aph cassette was cloned into the *Mlu*I and *Nru*I restriction sites of pCam-148 next to the NCTC11168 *htrA* gene. The resulting *htrA* complementation vector was called pSB-250. pSB-250 was then transformed into the *C. jejuni* NCTC11168Δ*htrA* deletion mutant (Boehm et al., 2012, 2013) and called NCTC11168Δ*htrA/htrA*. Correct integration of *htrA* in the *C. jejuni* chromosome was confirmed by PCR and standard sequencing. Expression of HtrA proteins was verified by Western blotting.

### Growth of *C. jejuni* strains on MH agar plates

*C. jejuni* NCTC11168 WT, NCTC11168Δ*htrA* and NCTC11168Δ*htrA/htrA* were grown overnight on Müller-Hinton (MH) agar plates at 37°C under microaerobic conditions using CampyGen gas packs (Oxoid, Wesel, Germany). Bacterial cells were harvested using brain heart infusion broth, and the OD_600_ was adjusted to 0.1. Subsequently, serial dilutions were made, and 10 μl volumes of the 10^−1^, 10^−2^, 10^−3^, 10^−4^, 10^−5^, and 10^−6^ dilutions were spotted onto three MH agar plates, which were incubated under microaerobic conditions using for 3 days at 42 or 44°C, or at 42°C in the presence of 18% O_2_ as described (Brøndsted et al., 2005). Experiments were repeated at least three times.

### Casein zymography

Bacterial lysates, culture supernatants or recombinant HtrA were separated under non-reducing conditions in gels containing casein. Subsequently, gels were renatured in 2.5% Triton-X-100 and equilibrated in developing buffer (Boehm et al., 2012; Hoy et al., 2012). Caseinolytic activity was visualized by staining with 0.5% Coomassie Blue R250.

### Mice

IL-10^−/−^ knockout mice (in C57BL/10 background, B10) were bred and maintained under specific pathogen-free (SPF) conditions in the facilities of the “Forschungsinstitut für Experimentelle Medizin” (FEM, Charité - Universitätsmedizin, Berlin, Germany). To eradicate the commensal gut flora, mice were transferred to sterile cages and treated by adding a mix of ampicillin (1 g/L; Ratiopharm), vancomycin (500 mg/L; Cell Pharm), ciprofloxacin (200 mg/L; Bayer Vital), imipenem (250 mg/L; MSD), and metronidazole (1 g/L; Fresenius) to the drinking water *ad libitum* starting at 3 weeks of age right after weaning (Heimesaat et al., 2006; Haag et al., 2012a). Age matched female mice were subjected to the quintuple antibiotic treatment for approximately 4 months before the infection experiments.

### *C. jejuni* infection of mice

Mice were infected with 10^9^ viable colony forming units (CFU) of the *C. jejuni* parental strain NCTC11168 WT strain or the isogenic mutant strain NCTC11168Δ*htrA* by gavage in a total volume of 0.3 mL PBS on 2 consecutive days (day 0 and 1) as described (Haag et al., 2012a,b).

### Clinical scoring

To assess clinical signs of *C. jejuni* induced infection on a daily basis, a standardized cumulative clinical score (maximum 12 points, addressing the occurrence of blood in feces (0 points: no blood; 2 points: microscopic detection of blood using Haemoccult, Beckman Coulter / PCD, Krefeld, Germany; 4 points: overt blood visible), diarrhea (0: formed feces; 2: pasty feces; 4: liquid feces), and the clinical aspect (0: normal; 2: ruffled fur, less locomotion; 4: isolation, severely compromised locomotion, pre-final aspect) was used (Haag et al., 2012a,b).

### Sampling procedures, determination of colonic length, and histopathology

Mice were sacrificed by isofluran treatment (Abbott, Germany). Cardiac blood and tissue samples from mesenteric lymph nodes (MLNs), spleen, liver, kidneys, and intestinal tract (duodenum, ileum, and colon) were removed under sterile conditions. Absolute large intestinal lengths were determined by measuring the distance from the ascending colon leaving the caecum to the rectum by a ruler and expressed in cm. Intestinal samples from each mouse were collected in parallel for histopathological, immunohistochemical, microbiological, and immunological analyses. Immunohistopathological changes were determined in samples derived from colon, liver and kidney that were immediately fixed in 5% formalin and embedded in paraffin. Sections (5 μm) were stained with hematoxylin and eosin (H&E), examined by light microscopy (magnification 100× and 400×) and histopathological changes quantitatively assessed applying respective histopathological scoring systems by two independent double-blinded investigators. In brief:

**Colonic Histopathology** (max. 4 points; according to Paclik et al., 2008): 0: no inflammation; 1: single isolated cell infiltrates within the mucosa,; no epithelial hyperplasia; 2: mild scattered to diffuse cell infiltrates within the mucosa and submucosa; mild epithelial hyperplasia; starting loss of goblet cells; 3: cell infiltrates within mucosa, submucosa, and sometimes transmural; epithelial hyperplasia; loss of goblet cells; 4: cell infiltrates within mucosa, submucosa, and transmural; severe inflammation; loss of goblet cells, loss of crypts; ulcerations; severe epithelial hyperplasia.
**Hepatic Histopathology** (max. 9 points; modified Ishak score, Ishak et al., 1995): Lobular inflammation: 0: normal; 1: minimal inflammation (few inflammatory infiltrates); 2: mild inflammation (increased inflammatory cells, but less pyknotic necrosis); 3: moderate inflammation (marked increase in inflammatory cells and lots of pyknotic necroses); 4: severe inflammation (necrosis); 5: severe inflammation (plus bridging necroses). Portal inflammation: 0: normal; 1: mild inflammation (<1/3 of portal tracts); 2: moderate inflammation (ca. 1/2 of portal tracts); 3: severe inflammation (>2/3 of portal tracts); 4: severe inflammation (plus portal inflammation disperse into parenchyma).
**Renal Histopathology** (max. 4 points; according to Appel et al., 1978): 0: normal glomerulus; 1: focal and mild hypercellularity (normal = 3 per segment); 2: multifocal and moderate hypercellularity with capillary dilatation and mild hyalinosis; 3: diffuse hypercellularity (>50% of the tuft) and capillary aneurysm; 4: extensive sclerosis/crescents (>3 cell layer), tuft obliteration, collapse.

### Immunohistochemistry

*In situ* immunohistochemical analysis of 5 μm thin colonic paraffine sections was performed as described previously (Heimesaat et al., 2010, 2013; Bereswill et al., 2011; Haag et al., 2012a,b). Primary antibodies against cleaved caspase-3 (Asp175, Cell Signaling, USA, 1:200), Ki67 (TEC3, Dako, Denmark, 1:100), CD3 (M-20, Santa Cruz, dilution 1:1000), myeloperoxidase-7 (MPO-7, # A0398, Dako, 1:500), F4/80 (# 14-4801, clone BM8, eBioscience, 1:50), FOXP3 (FJK-16s, eBioscience, 1:100), and B220 (eBioscience, San Diego, CA, USA, 1:200) were used. For each animal, the average number of positively stained cells within at least six high power fields (HPF, 0.287 mm^2^; 400× magnification) were determined microscopically by two independent double-blinded investigators and subjected to statistical analysis as indicated below.

### Quantitative analysis of *C. jejuni* and bacterial translocation into other organs

Live *C. jejuni* were detected at time of necropsy (day 6 p.i.) in luminal samples taken from the duodenum, ileum, or colon diluted in sterile PBS by culture as described earlier (Bereswill et al., 2011). To quantify bacterial translocation into different organs, MLNs, spleen, liver, and kidney were homogenized in sterile PBS and analyzed by cultivating in dilution series on karmali agar (Oxoid, Wesel, Germany) in a microaerobic atmosphere at 37°C for at least 48 h (Heimesaat et al., 2013). In addition, 0.5 mL of cardiac blood was streaked out immediately on karmali agar. The respective weights of luminal fecal or tissue samples were determined by the difference of the sample weights before and after asservation.

### Cytokine detection in serum samples and culture supernatants of *ex vivo* biopsies taken from colon, mesenteric lymph nodes, spleen, liver, and kidney

Colonic biopsies and kidneys were cut longitudinally and the former washed in PBS. MLNs or strips of approximately 1 cm^2^ colon and liver tissue and additionally half of spleen and kidney were placed in 24-flat-bottom well culture plates (Nunc, Wiesbaden, Germany) containing 500 μL serum-free RPMI 1640 medium supplemented with penicillin (100 U/mL) and streptomycin (100 μg/mL; PAA Laboratories). After 18 h of incubation at 37°C, culture supernatants as well as serum samples were analyzed for IFN-γ, TNF-α, and IL-6 by the Mouse Inflammation Cytometric Bead Assay (CBA; BD Biosciences) on a BD FACS Canto II flow cytometer (BD Biosciences). Nitric oxide (NO) was determined by Griess reaction as described earlier (Heimesaat et al., 2006).

### Quantitative real-time PCR (Qrt-PCR)

RNA was isolated from colonic tissues using the RNeasy Mini Kit (Qiagen). mRNA was reversed transcribed and analyzed in triplicate assays by TaqMan PCR using a sequence detection system (ABI Prism 7700; Applied Biosystems) as described previously (Wolk et al., 2002; Munoz et al., 2009). For detection of murine MUC-2 assays including double-fluorescent probes in combination with assays for the mouse housekeeping gene hypoxanthine phosphoribosyltransferase (HPRT) were purchased from Applied Biosystems. Expression levels were calculated relative to the HPRT expression.

### Antibodies and western blotting

*C. jejuni* cell pellets were lysed and proteins were separated by SDS-PAGE (Krause-Gruszczynska et al., 2007b; Wiedemann et al., 2012). The polyclonal rabbit α-HtrA antibody was raised against a conserved peptide corresponding to amino acid (aa) residues 288–301: C-QGDTKKAYKNQEGA. The α-CiaB antibody was generated against the epitope 597–610 (C-EIDNSGEFERYKKK) and the α-MOMP antibody against aa residues 400–413 (C-NLDQGVNTNESADH) in the corresponding proteins, respectively. All three peptides were conjugated to *Limulus polyphemus* haemocyanin carrier protein, and two rabbits each were immunized by Biogenes GmbH (Berlin, Germany) using standard protocols (Tegtmeyer et al., 2013). The resulting antiserum was affinity-purified and the specificity against the proteins in *C. jejuni* was confirmed by Western blotting (Tegtmeyer et al., 2011; Backert and Hofreuter, 2013). Horseradish peroxidase-conjugated anti-rabbit polyvalent sheep immunoglobulin was used as secondary antibody (DAKO Denmark A/S, DK-2600 Glostrup, Denmark). Blots were developed with ECL Plus Western blot reagents (GE Healthcare, UK limited Amersham Place, UK) as described (Conradi et al., 2012; Hirsch et al., 2012).

### Statistical analysis

Mean values, medians, and levels of significance were determined using Mann-Whitney-U-test. Two-sided probability (*P*) values ≤ 0.05 were considered significant. All experiments were repeated various times as indicated in the corresponding figure legends.

## Results

### HtrA does not affect the colonization capacity of *C. jejuni* in gnotobiotic IL-10^−/−^ mice

**Figure 1:**
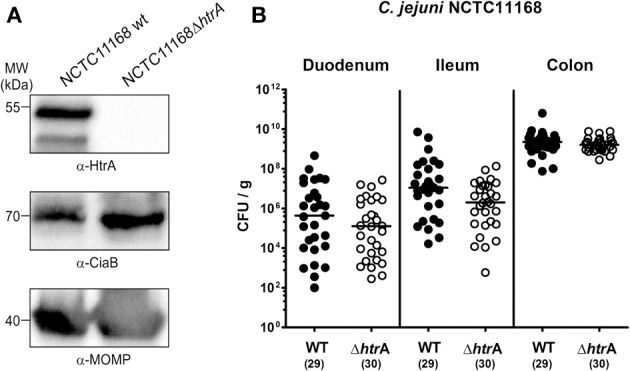
***C. jejuni* colonization along the intestinal tract of gnotobiotic IL-10^−/−^ mice is independent of *htrA* gene expression. (A)** Control Western blots for the expression of indicated proteins in the two *C. jejuni* strains used in this infection study. **(B)** Gnotobiotic IL-10^−/−^ mice were generated by broad-spectrum antibiotic treatment and perorally infected with *C. jejuni* NCTC11168 wild-type strain (WT, closed circles) or mutant strain NCTC11168Δ*htrA* (Δ*htrA*, open circles). The pathogen densities in distinct compartments of the intestinal tract were determined by quantification of live *C. jejuni* in luminal samples taken from duodenum, ileum, and colon at day 6 p.i. by cultural analysis (CFU, colony forming units). Medians (black bars) are indicated and numbers of analyzed animals given in parentheses. Data shown were combined from five independent experiments.

In order to eradicate the chronic colitogenic stimulus derived from the commensal intestinal microbiota, IL-10^−/−^ mice were pre-treated with a quintuple antibiotic regimen for approximately 4 months starting immediately after weaning (Haag et al., 2012a). The resulting gnotobiotic IL-10^−/−^ mice were then perorally infected with 10^9^ CFU of either *C. jejuni* NCTC11168 WT or isogenic *htrA* mutant (NCTC11168Δ*htrA*) strain, each grown to stationary phase on 2 consecutive days (day 0 and 1). Control blots demonstrate that equal amounts of *C. jejuni* were infected per sample and HtrA is not expressed in the Δ*htrA* mutant as expected (Figure 1A). We also confirmed that our Δ*htrA* mutant is non-polar because genetic complementation experiments with the corresponding WT gene restored (i) expression of proteolytically active HtrA multimers (Figures S1A,B), (ii) *C. jejuni* growth at high temperature (44°C) (Figures S2A,B) and (iii) growth under high oxygen stress conditions (Figures S2A,C). Six days following peroral challenge all mice harbored comparable pathogen loads with either strain in the duodenum, ileum and colon, which is indicative for an uncompromised colonization capacity of the Δ*htrA* mutant strain *in vivo* (Figure 1B). After 6 days 12.5% of IL-10^−/−^ mice infected with either the Δ*htrA* mutant or the *C. jejuni* WT strain contained viable pathogens also in MLNs as shown by culture. Notably, bacterial presence in extra-intestinal compartments such as spleen, liver, kidney, or blood could not be observed with either strain based on CFU determination (data not shown).

### Impact of HtrA on acute enterocolitis in *C. jejuni* infected gnotobiotic IL-10^−/−^ mice

**Figure 2:**
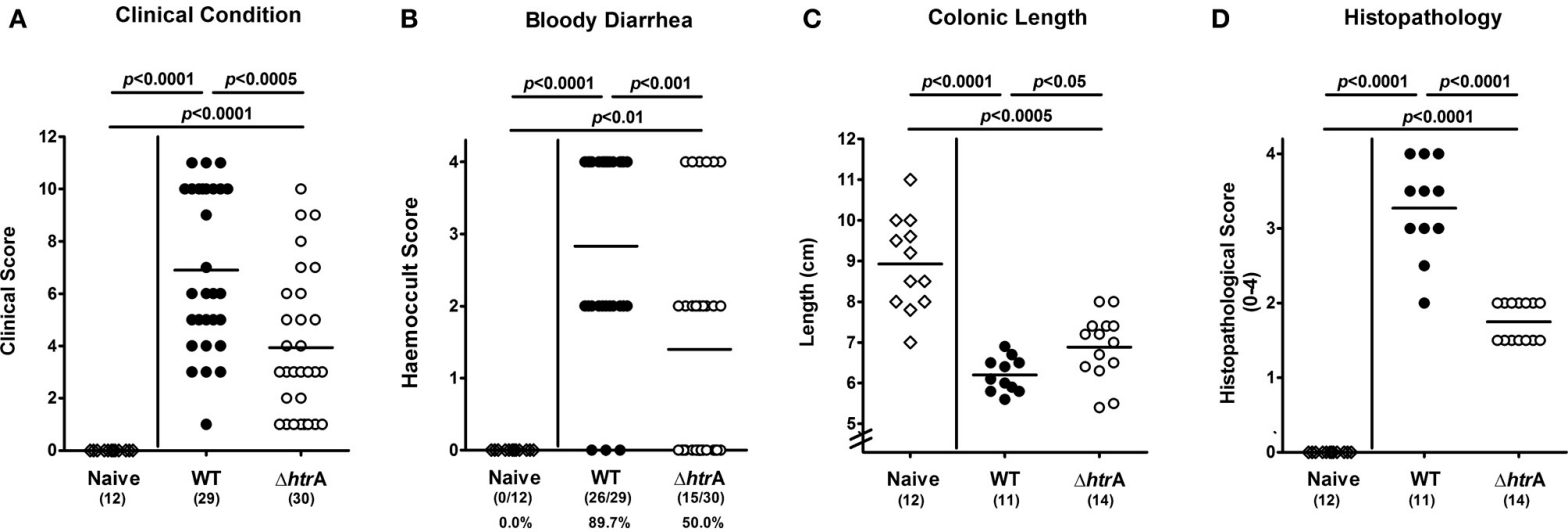
**Role of *C. jejuni htrA* gene in acute enterocolitis following infection of gnotobiotic IL-10^−/−^ mice.** Six days following *C. jejuni* infection **(A)** disease activity and **(B)** occurrence of blood in fecal samples was assessed by applying respective standardized clinical scoring system. Furthermore, **(C)** large intestinal lengths (in cm) were measured and **(D)** histopathological changes applying a standardized histopathological score in H&E-stained colonic paraffin sections were determined at necropsy. Means (black bars), levels of significance (*P*-values) determined by the Mann-Whitney-U-test, and numbers of analyzed animals (in parentheses) or absolute and relative (in %) numbers of positive samples out of the total number are indicated. Data shown were pooled from three independent experiments.

Six days p.i. with the *C. jejuni* NCTC11168 WT strain, gnotobiotic IL-10^−/−^ mice were decisively compromised by acute enterocolitis as indicated by wasting clinical symptoms, diarrhea and occurrence of blood in liquid feces in up to 90% of cases (Figures 2A,B). In contrast, mice infected with the Δ*htrA* mutant displayed significantly less severe clinical symptoms (*p* < 0.0005; Figure 2A) and lower frequency of bloody diarrhea as compared to controls infected with the WT strain (50.0 vs. 89.7%, respectively; *p* < 0.001, Figure 2B). Given that intestinal inflammation results in a significant shortening of the intestines (Heimesaat et al., 2006; Bereswill et al., 2011; Haag et al., 2012a), we further assessed the colonic lengths upon *C. jejuni* infection. At day 6 p.i., IL-10^−/−^ mice infected with the Δ*htrA* mutant displayed longer colons as compared to WT strain infected control animals (*p* < 0.05; Figure 2C). These results provide first evidence that HtrA aggravates the inflammatory outcome of *C. jejuni* infection. This was further confirmed by histopathological analysis of paraffin embedded colonic sections. Microscopical investigations of H&E-stained tissues revealed that Δ*htrA* mutant infected mice displayed significantly lower histopathological scores as compared to mice infected with the *C. jejuni* WT strain at day 6 p.i. (*p* < 0.0001, Figure 2D). The Δ*htrA* mutant induced rather mild inflammatory changes whereas *C. jejuni* WT strain infected mice exhibited acute enterocolitis characterized by ulcerations of and bleeding into the colonic mucosa as well as by diffuse mucosal and submucosal leukocytic infiltrates, loss of goblet cells and crypt drop-outs (Figure 2D).

### HtrA triggers *C. jejuni*-mediated intestinal immune responses

**Figure 3:**
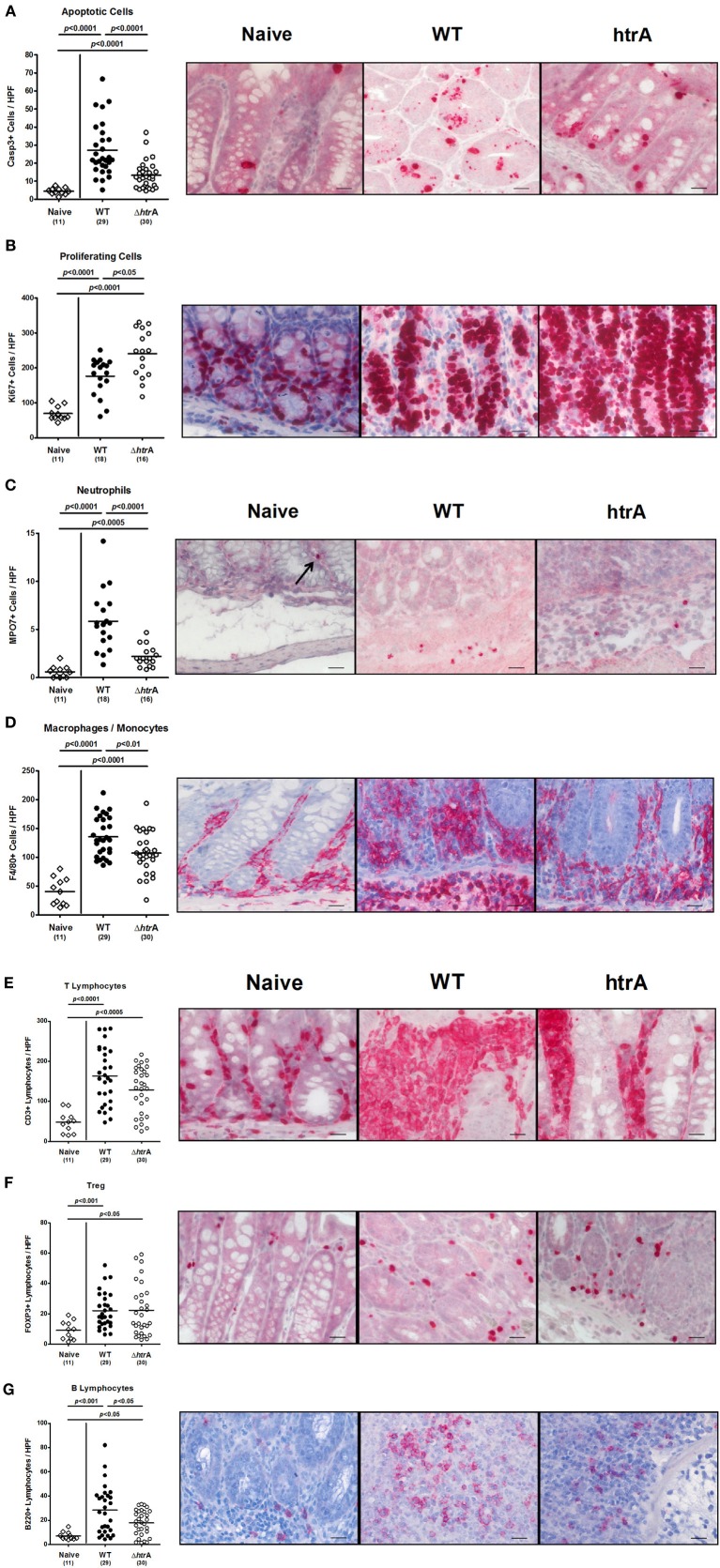
**Colonic inflammatory, regenerative, and immune cell responses in gnotobiotic IL-10^−/−^ mice following infection with wild-type and Δ*htrA* mutant *C. jejuni*.** The average numbers of apoptotic cells [positive for caspase-3, **(A)**], proliferating cells [positive for Ki67, **(B)**], neutrophils [positive for MPO7, **(C)**], macrophages and monocytes [positive for F4/80, **(D)**], T lymphocytes [positive for CD3, **(E)**], regulatory T cells [Treg, positive for FOXP3; **(F)**], and B lymphocytes [positive for B220, **(G)**] from at least six high power fields (HPF, 400× magnification) per animal were determined microscopically in immunohistochemically stained colon sections at day 6 p.i. Data were pooled from five independent experiments. Representative photomicrographs of respectively stained cells are shown (400× magnification, scale bar 20 μm).

The role of *C. jejuni* HtrA in intestinal inflammation was next assessed by microscopical quantification of apoptotic and proliferating cells as well as infiltrating immune cells. This was achieved by specific immunohistochemical stainings of colonic paraffin sections. Six days following *C. jejuni* WT strain infection, gnotobiotic IL-10^−/−^ mice displayed a multifold increase of apoptotic cells, neutrophils, macrophages and monocytes, B and T lymphocytes as well as regulatory T cells (Treg) in the colonic mucosa and lamina propria (*p* < 0.001−0.0001 as compared to naive control animals; Figure 3). The induction of apoptosis and the increase in inflammatory immune cells, however, were significantly less pronounced in animals infected with the *C. jejuni* Δ*htrA* mutant except for the T cell and Treg populations (*p* < 0.05−0.0001 as compared to animals infected with the *C. jejuni* WT strain; Figure 3). Given that Ki67 comprises a nuclear protein that is associated with and necessary for cellular proliferation (Scholzen and Gerdes, 2000), we stained colonic paraffin sections against Ki67 to detect proliferative measures of the colonic epithelium counteracting cellular destruction during the inflammatory process. Interestingly, the Δ*htrA* mutant induced significantly higher numbers of Ki67^+^ proliferating cells as compared to the WT strain (*p* < 0.05; Figure 3B).

**Figure 4:**
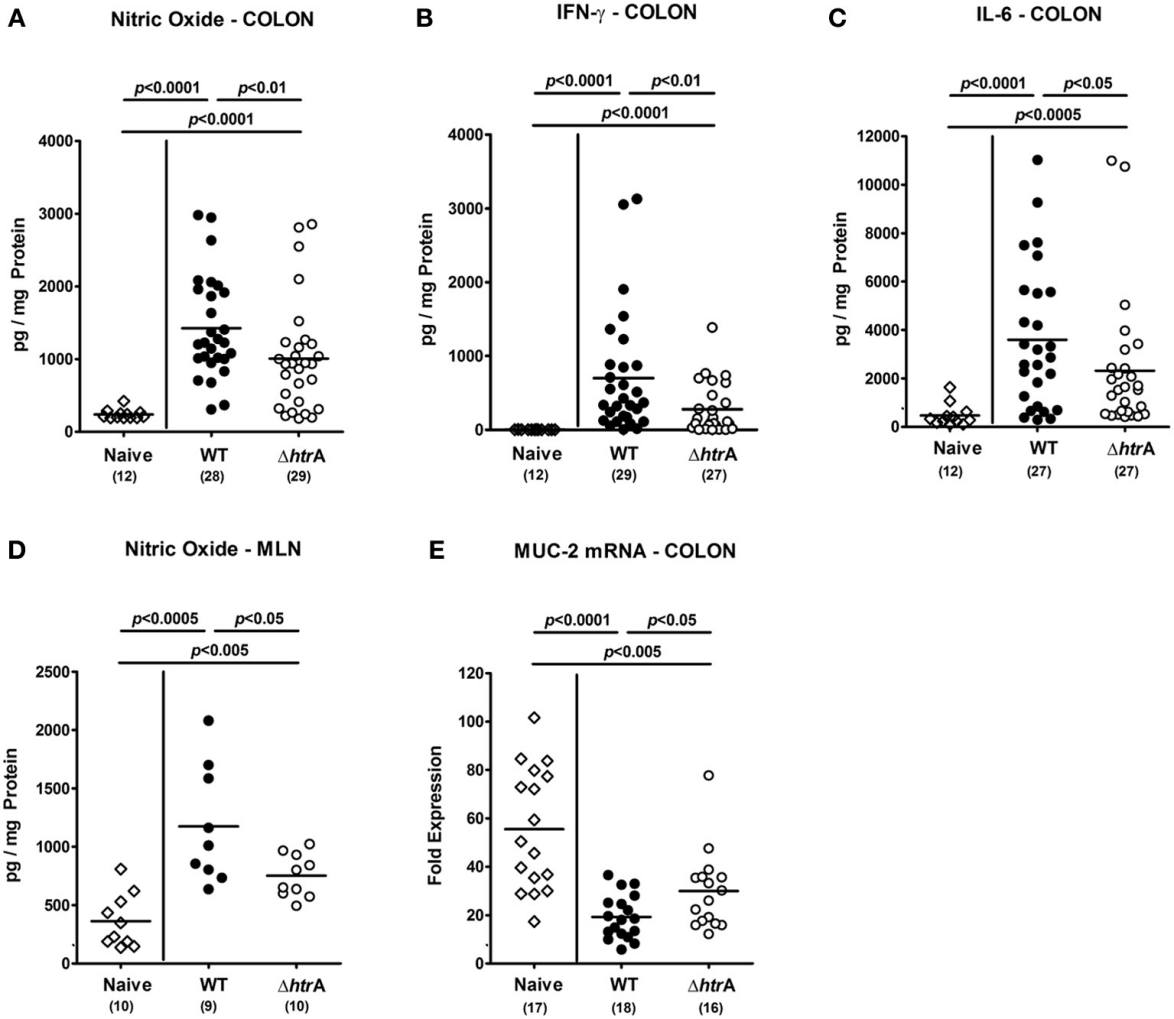
**Importance of *C. jejuni htrA* gene expression in intestinal pro-inflammatory cytokine responses and mucin-2 expression induced during infection of gnotobiotic IL-10^−/−^ mice.** Colonic **(A)** nitric oxide, **(B)** IFN-γ, and **(C)** IL-6 levels, **(D)** nitric oxide concentrations in mesenteric lymph nodes (MLN), and **(E)** large intestinal mucin-2 (MUC-2) mRNA expression levels were determined in respective *ex vivo* biopsies taken at day 6 p.i. Data shown were combined from three independent experiments.

The *C. jejuni* induced colonic immune cell responses were accompanied by increased expression of pro-inflammatory cytokines in the large intestine. Until day 6 p.i., levels, of IFN-γ and IL-6 and, in addition, NO were multifold increased in colonic *ex vivo* biopsies upon *C. jejuni* infection. Cytokines increased to a significantly lower extent in gnotobiotic IL-10^−/−^ mice infected with the Δ*htrA* mutant as compared to WT strain infected mice (*p* < 0.05−0.01; Figures 4A–C). In addition, *C. jejuni* infected mice displayed increased NO secretion into draining MLNs at day 6 p.i. as compared to uninfected controls. This increase, however, was significantly lower in mice infected with the Δ*htrA* mutant vs. the parental WT strain infected animals (*p* < 0.05; Figure 4D).

Given that a proper mucus layer is a pivotal barrier protecting the intestinal epithelium from intestinal pathogens, we next determined the mRNA expression levels of mucin-2 (MUC-2), which is mainly secreted from goblet cells in the epithelial lining of the large intestine (Allen et al., 1998; Naughton et al., 2014). Six days following *C. jejuni* infection of IL-10^−/−^ mice, colonic mucin-2 mRNA was significantly downregulated by *C. jejuni* WT strain, but to a lesser extent by the Δ*htrA* knockout mutant (*p* < 0.05; Figure 4E). Taken together, the less severe clinical, histopathological and inflammatory outcome of enterocolitis in mice infected with the *C. jejuni* Δ*htrA* mutant strain was paralleled by a higher mucin-2 expression level as compared to control animals infected with the parental strain.

### HtrA is also involved in the induction of extra-intestinal pro-inflammatory immune responses in *C. jejuni* infected gnotobiotic IL-10^−/−^ mice

**Figure 5:**
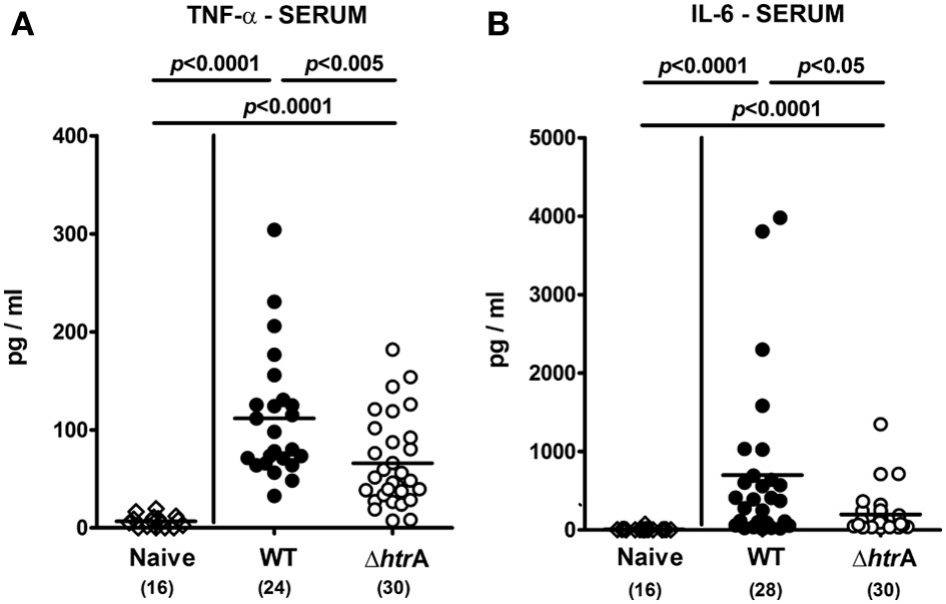
**Systemic pro-inflammatory cytokine responses following *C. jejuni* infection of gnotobiotic IL-10^−/−^ mice. (A)** TNF-α and **(B)** IL-6 levels were determined in serum samples taken at day 6 p.i. Data shown were pooled from five independent experiments.

**Figure 6:**
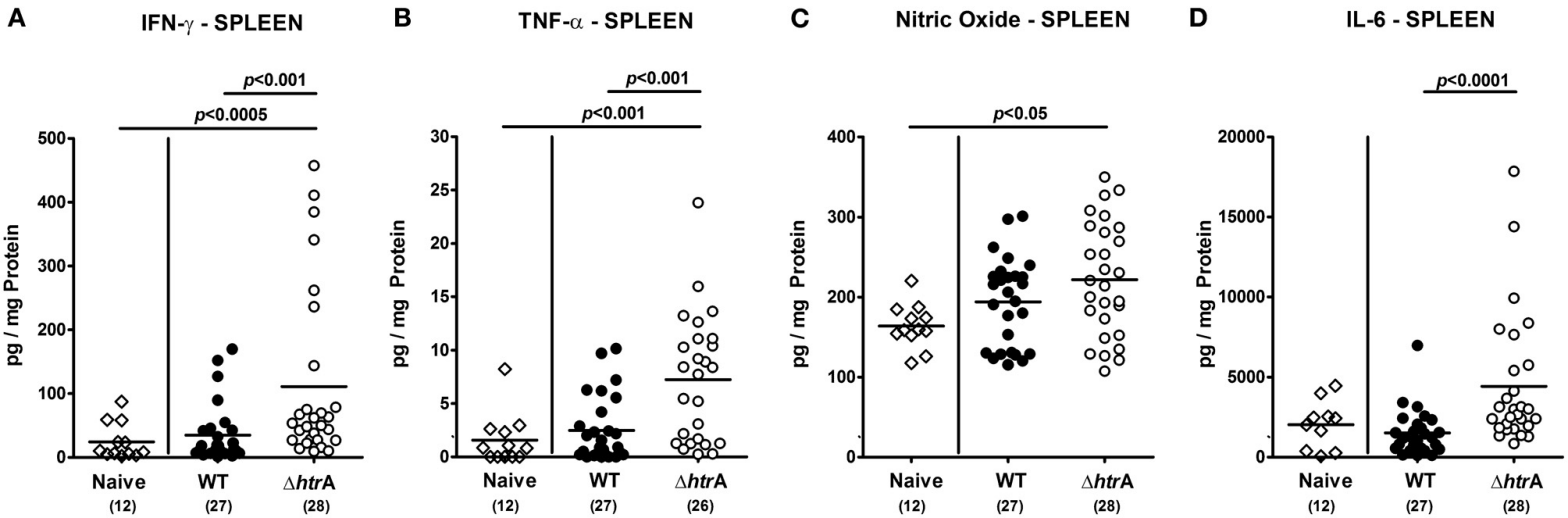
**Pro-inflammatory cytokine responses in spleens following *C. jejuni* infection of gnotobiotic IL-10^−/−^ mice.** Splenic **(A)** IFN-γ, **(B)** TNF-α, **(C)** nitric oxide, and **(D)** IL-6 levels were determined in supernatants of *ex vivo* biopsies taken at day 6 p.i. Data shown were pooled from five independent experiments.

We next investigated potential systemic pro-inflammatory immune responses upon *C. jejuni* infection. At day 6 p.i. with either strain, TNF-α and IL-6 serum levels were increased as compared to naïve controls, but less distinctly in Δ*htrA* mutant strain infected mice (*p* < 0.005 and *p* < 0.05, respectively; Figure 5). Hence, *C. jejuni* htrA knockout mutation not only reduces intestinal but also systemic inflammatory responses upon infection. Unexpectedly, 6 days following infection with the Δ*htrA* gene mutant but not the WT strain, IFN-γ, TNF-α, and NO levels were up-regulated in splenic *ex vivo* biopsies (*p* < 0.05−0.0005; Figures 6A–C). Furthermore, secretion of IL-6 into spleens were higher following Δ*htrA* mutant infection as compared to the parental strain at day 6 p.i. (*p* < 0.0001, Figure 6D).

**Figure 7:**
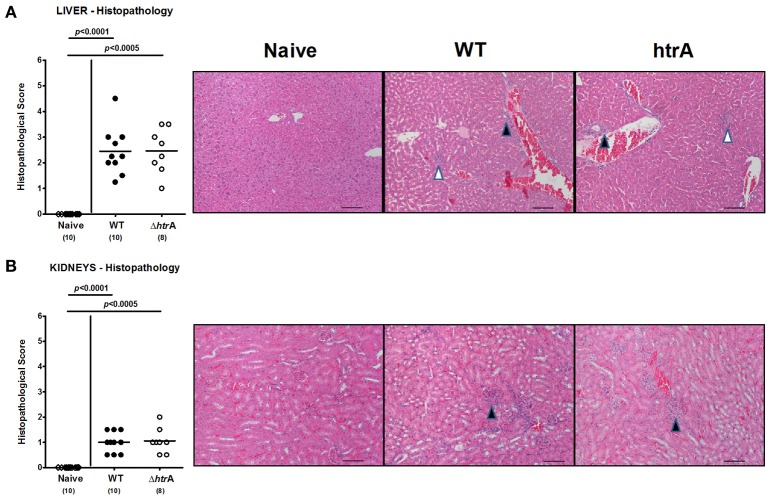
**Extra-intestinal immunopathological sequelae following *C. jejuni* infection of gnotobiotic IL-10^−/−^ mice.** Six days following *C. jejuni* infection extra-intestinal immunopathological sequelae were assessed in H&E-stained paraffin sections of **(A)** liver and **(B)** kidneys by applying respective standardized clinical scoring system and illustrated by representative photomicrographs (400× magnification, scale bar 20 μm). In liver sections, black arrow heads indicate portal and white arrow head lobular inflammatory infiltrates. In kidney sections, black arrow heads point to focal hypercellularity. Data shown were combined from three independent experiments.

We have recently shown that following long-term *C. jejuni* infection (i.e., more than 100 days p.i.) conventionally colonized infant mice exhibited pro-inflammatory immune responses at extra-intestinal locations such as liver and kidneys (Haag et al., 2012b; Heimesaat et al., 2013). We were therefore interested to investigate whether extra-intestinal sequelae might arise even after relatively short-term *C. jejuni* infection of gnotobiotic IL-10^−/−^ mice suffering from acute enterocolitis. To address this idea, we assessed inflammatory changes in H&E-stained paraffin sections of liver and kidneys. Six days following *C. jejuni* infection with either strain mice displayed only minimal to mild lobular and/or portal inflammatory infiltrates in livers and mild focal hypercellularity in kidneys (Figure 7).

**Figure 8:**
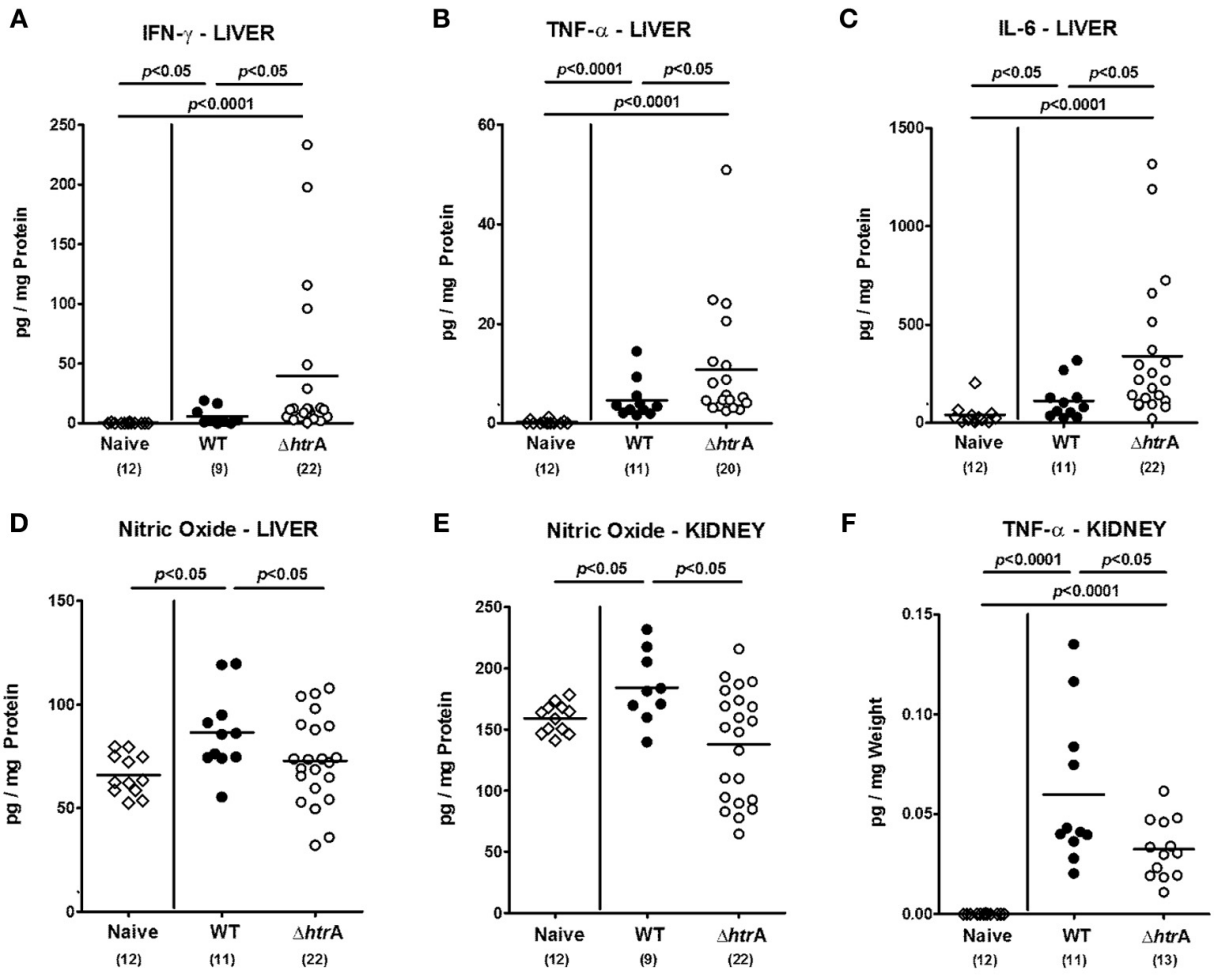
**Pro-inflammatory cytokine responses in livers and kidneys of gnotobiotic IL-10^−/−^ mice infected with *C. jejuni*.** Hepatic **(A)** IFN-γ, **(B)** TNF-α, **(C)** IL-6, **(D)** nitric oxide levels, and renal **(E)** nitric oxide, and **(F)** TNF-α levels were determined in supernatants of *ex vivo* biopsies taken at day 6 p.i. Data shown were pooled from at least three independent experiments.

We next determined pro-inflammatory cytokine responses in *ex vivo* biopsies from liver and kidneys upon *C. jejuni* infection of gnotobiotic IL-10^−/−^ mice. Six days following *C. jejuni* infection hepatic IFN-γ, TNF-α, and IL-6 concentrations increased (*p* < 0.05−0.0001; Figures 8A–C), but to even higher levels upon Δ*htrA* knockout mutant as compared to parental *C. jejuni* WT strain infection (*p* < 0.05; Figures 8A–C). Notably, the standard deviation in the cohort infected with the Δ*htrA* gene knockout mutant was rather high. Interestingly, hepatic NO levels increased 6 days following WT infection, but not during Δ*htrA* mutant strain infection (*p* < 0.05; Figure 8D), which helds also true for NO secretion in *ex vivo* biopsies taken from kidneys (*p* < 0.05; Figure 8E). Furthermore, TNF-α levels increased in kidneys of *C. jejuni* infected IL-10^−/−^ mice (*p* < 0.0001; Figure 8F), but less distinctly in the Δ*htrA* knockout mutant infected group (*p* < 0.05; Figure 8F). Taken together, even as early as 6 days following *C. jejuni* infection pro-inflammatory immune responses could be detected in extra-intestinal compartments such as liver and kidneys that were even more pronounced in livers, but less distinct in kidneys of Δ*htrA* mutant as compared to parental WT strain infected IL-10^−/−^ mice suffering from acute enterocolitis.

## Discussion

The mucosa in the intestine of mammals forms a tight barrier, which protects against commensals and other microbes present in the intestinal lumen. Previous infection studies in mice have revealed that mutation of the serine protease HtrA in a number of pathogenic bacteria results in attenuated virulence properties (Li et al., 1996; Humphreys et al., 1999; Wilson et al., 2006; Yuan et al., 2008; Lewis et al., 2009; De Stoppelaar et al., 2013). Bacterial HtrA may be involved in degradation of damaged bacterial proteins that accumulate during the hostile conditions in the macrophages and accordingly, *htrA* mutants of the facultative intracellular *Salmonella typhimurium, Listeria monocytogenes*, and *Yersinia enterocolitica* are all sensitive to oxidative agents and show reduced survival rates in macrophages (Bäumler et al., 1994; Yamamoto et al., 1996; Wilson et al., 2006). The individual contribution of chaperone and protease activity of HtrA to bacterial survival and multiplication in the host is difficult to dissect. However, for *S. typhimurium*, both activities appear to be required for proper systemic infection in mice (Lewis et al., 2009). Interestingly, after oral infection with an *htrA* mutant, the pathogen cannot translocate accurately from the Peyer's patches to other organs. In contrast, intravenous infection of mice resulted in full colonization of livers and spleens by the *S. typhimurium htrA* mutant, suggesting that HtrA is required to overcome the intestinal barrier (Humphreys et al., 1999). Expression of HtrA is also important for cell-to-cell spread of *Shigella flexneri* (Purdy et al., 2002, 2007). Previous *in vitro* infection studies with *C. jejuni* have indicated that inactivation of *htrA* reduced the bacterial adherence to cultured epithelial cells (Brøndsted et al., 2005). In addition, *C. jejuni htrA* mutants exhibited reduced invasion rates (Novik et al., 2010; Bæk et al., 2011a). Interestingly, HtrA chaperone activity appears to be important for efficient binding of *C. jejuni* to epithelial cells, while the HtrA protease activity seems required for maximal host cell entry after the bacteria have adhered to them (Bæk et al., 2011a). Under laboratory conditions *in vitro*, HtrA chaperone activity is necessary for growth of *C. jejuni* at high temperatures or under oxidative stress conditions, whereas HtrA protease activity is only essential during severe stress (Bæk et al., 2011b).

We have recently shown in a series of *in vitro* experiments that HtrA secreted by *C. jejuni* constitutes a novel bacterial virulence determinant, which opens cell-to-cell junctions through cleavage of E-cadherin and probably other host factors (Boehm et al., 2012, 2013; Hoy et al., 2012). In the present study, we investigated for the first time the impact of the *htrA* gene in *C. jejuni-*induced immunopathology *in vivo* and applied the acute *C. jejuni* infection model of gnotobiotic IL-10^−/−^ mice. To prevent conventionally colonized IL-10^−/−^ mice from chronic colitis exerted by antigenic stimuli through the conventional intestinal microbiota, mice were pre-treated for 4 months with a quintuple antibiotic regimen starting immediately after weaning (Haag et al., 2012a). Six days following peroral infection with *C. jejuni*, mice harbored high intestinal loads of the non-polar knockout mutant strain NCTC11168Δ*htrA*, which were comparable to those detected in mice upon infection with the parental WT strain NCTC11168. Hence, inactivation of the *htrA* gene did not down-regulate the overall high colonization capacity of *C. jejuni* in gnotobiotic mice. At first sight, these data seem to contradict a previous report where HtrA chaperone activity was shown to be required for efficient binding of *C. jejuni* to cultured INT-407 epithelial cells (Bæk et al., 2011a). However, INT-407 cells do not form polarized cell layers, while polarized epithelial cell layers are found in the intestine of live mice. Thus, the receptor availability in both systems is certainly different, which could be one reason to explain these findings.

Importantly, gnotobiotic IL-10^−/−^ mice infected with the parental WT strain were severely compromised and developed ulcerative enterocolitis with bloody diarrhea and wasting symptoms, hence mimicking severe campylobacteriosis in immuno-compromised patients (Haag et al., 2012a). Remarkably, at day 6 p.i. ulcerative enterocolitis was less distinct in mice infected with the Δ*htrA* mutant, which displayed significantly less severe immunopathology in the intestinal tract as compared to mice infected with the *C. jejuni* WT strain. Interestingly, Δ*htrA* mutant infected mice exhibited higher Ki67^+^ proliferating cell numbers in the colonic mucosa as compared to WT strain infected controls. This might be indicative for up-regulated compensatory properties in order to counteract cell destruction during immunopathology. Furthermore, not only local, but even systemic immune responses were less pronounced upon Δ*htrA* mutant infection as indicated by significant lower serum levels of pro-inflammatory cytokines such as TNF-α and IL-6 in Δ*htrA* mutant as compared to WT strain infected mice. The amelioration of devastating enteric and systemic disease, as in this hyper-acute model system, underlines the biological relevance of individual *C. jejuni* virulence factors *in vivo*. Furthermore, in the present and previous infection studies with conventional infant, germfree or with human microbiota re-associated germfree WT mice (Bereswill et al., 2011; Haag et al., 2012b), translocation of viable *C. jejuni* of either strain from the intestine to the MLNs could be observed in a subset of animals, whereas no bacterial CFUs could be cultured from extra-intestinal locations such as spleen, liver, kidney, or cardiac blood. This is good agreement with the observation of very rare cases of extra-intestinal *C. jejuni*-associated disease affecting the liver, lung, heart, or spleen that have been reported in immunocompromised patients suffering from *C. jejuni* bacteremia (Pigrau et al., 1997; Tee and Mijch, 1998; Crushell et al., 2004) and previous infection studies of isolator-raised germfree mice (Fauchère et al., 1985). Whereas viable *C. jejuni* could be cultured from MLNs of infected mice more than 3 weeks p.i., the pathogen was cleared from the circulation and extra-intestinal organs such as liver and spleen within 24 h. The authors proposed that non-specific bactericidal factors such as complement or phagocytic cells might have been responsible for rapid pathogenic clearance. However, the histopathological sequelae within the respective organs were not reported (Fauchère et al., 1985). Surprisingly, in the present study histopathological analysis revealed rather mild inflammatory changes in extra-intestinal organs such as liver and kidneys that did not differ 6 days p.i. with either *C. jejuni* strain. In gnotobiotic WT mice, however, which can be readily colonized by *C. jejuni*, we did not observe any overt extra-intestinal histopathological changes in respective H&E stained paraffin sections by day 42 p.i. (unpublished observations). It is therefore most likely that a significant intestinal pro-inflammatory scenario (as seen in the gnotobiotic IL-10^−/−^ mouse model) is a prerequisite for subsequently induced extra-intestinal inflammatory responses as shown in the present study. In line with this, in another infection model, 3 weeks old infant mice (harboring a conventional microbiota) were infected with *C. jejuni* B2 strain immediately after weaning and exhibited histopathological sequelae in liver, kidneys, lungs, and cardiac muscle more than 100 days following *C. jejuni* infection (Haag et al., 2012b). Interestingly, mice were asymptomatic *C. jejuni* carriers until day 103 p.i. and the extra-intestinal organs were exclusively *C. jejuni* culture negative (Haag et al., 2012b). Furthermore, the vast majority of inflammatory cells that had accumulated in the respective extra-intestinal organs comprised CD3^+^ T lymphocytes (Heimesaat et al., 2013). It is highly likely that pro-inflammatory immune cells have been attracted to the site of infection and cleared the pathogen early in the course of infection and reside further in the respective organ. This would explain the sterile inflammatory responses in liver and kidneys, both in less than 1 week (this study) and more than 3 months p.i. (Haag et al., 2012b; Heimesaat et al., 2013). Hence, the influx of pro-inflammatory immune cells upon even short-term *C. jejuni* infection might explain the increased levels of pro-inflammatory cytokines such as IFN-γ, IL-6, TNF-α, and NO detected in livers and the latter two in kidneys at day 6 p.i. Like in the colon, renal TNF-α and NO as well as hepatic NO levels were significantly lower in Δ*htrA* knockout mutant as compared to parental strain infected gnotobiotic IL-10^−/−^ mice at day 6 p.i. Unexpectedly, IFN-γ, IL-6, and TNF-α concentrations in spleen and liver were even higher 6 days following Δ*htrA* knockout mutant as compared to parental WT strain infection. Notably, the standard deviations in the cohorts infected with the Δ*htrA* gene knockout mutant were rather high, which held true for individual experiments as well as for pooled data sets. Hence, the individual variabilities upon infection needs to take into account when judging for the biological relevance of the observed effects in the absence of *htrA*. In line with these unexpected data derived from livers of infected mice, splenic IFN-γ, IL-6, and TNF-α levels increased only upon Δ*htrA* knockout mutant, but not parental WT strain infection when compared to naive controls. It is therefore tempting to speculate whether in the course of the observed systemic immune responses more immune cells (such as dendritic cells and/or lymphocytes) might have been activated/imprinted in the spleen by the *C. jejuni* Δ*htrA* mutant present in the intestine as compared to parental WT strain infection or stimulated by distinct (so far unknown) circulating soluble bacterial factors derived from the pathogen which in turn could counteract intestinal disease. Further studies need to unravel this fascinating infection phenomenon.

Taken together, *C. jejuni* is one of the most important zoonotic pathogens causing food-borne gastroenteritis and potentially more severe diseases. Crossing the intestinal epithelial barrier and host cell invasion by *C. jejuni* are considered to constitute primary reasons of gut tissue damage in humans. However, the molecular mechanisms as well as major bacterial and host cell factors involved in these activities are poorly understood. Using the IL-10^−/−^ knockout mouse infection model system, the results presented in this study demonstrate for the first time that the *C. jejuni* HtrA serine protease is a novel virulence factor which aggravates enterocolitis *in situ* by causing a substantial amount of cell damage, aggravation of intestinal apoptosis and inflammation upon *C. jejuni* infection *in vivo* accompanied by significant systemic pro-inflammatory immune responses. These observations are in line with our earlier *in vitro* studies showing that HtrA of *C. jejuni* targets epithelial E-cadherin-based cell-to-cell junctions (Boehm et al., 2012, 2013; Hoy et al., 2012). Future work should address important questions such as how *C. jejuni* can trigger HtrA secretion into the extracellular space, to identify E-cadherin cleavage sites by HtrA and to search for novel host cell targets involved in the above discovered activities *in vivo*. We further demonstrated non-polarity of the Δ*htrA* knockout mutant strain by complementation of phenotypes *in vitro*; however, this does not warranty non-polarity of the mutation *in vivo*. The investigation of epithelium-bound or internalized *C. jejuni* in ongoing studies will further complete our understanding of the complex interactions of HtrA with the epithelium.

## Financial disclosure, grant support

The funders had no role in study design, data collection and analysis, decision to publish, or preparation of the manuscript.

## Author contributions

Conceived and designed the experiments: Markus M. Heimesaat, André Fischer, Marie Alutis, Steffen Backert, Stefan Bereswill. Performed the experiments: Markus M. Heimesaat, André Fischer, Marie Alutis, Ursula Grundmann. Analyzed the data: Markus M. Heimesaat, André Fischer, Marie Alutis, Anja A. Kühl, Manja Böhm, Nicole Tegtmeyer, Steffen Backert, Stefan Bereswill. Contributed reagents/materials/analysis tolls: Ulf B. Göbel, Manja Böhm, Nicole Tegtmeyer, Anja A. Kühl. Wrote the paper: Markus M. Heimesaat, Anja A. Kühl, André Fischer, Steffen Backert, Stefan Bereswill.

### Conflict of interest statement

The authors declare that the research was conducted in the absence of any commercial or financial relationships that could be construed as a potential conflict of interest.

## Supplementary material

Figure S1 **Genetic complementation of HtrA reveals that the *C. jejuni* NCTC11168 Δ*htrA* mutant is non-polar. (A)** Expression of HtrA proteins by *C. jejuni* NCTC11168 wild-type (WT), NCTC11168Δ*htrA* mutant and complemented NCTC11168Δ*htrA/htrA* were investigated by Western blotting using an α-HtrA antibody. As control, equal amounts of protein per sample were confirmed by immunoblotting using the α-MOMP antibody. **(B)** Analysis of protease activities in all three indicated strains by casein zymography. The position of proteolytically active multimeric HtrA proteins is indicated with arrows.

Figure S2 **Effect of high temperature and oxygen concentration on growth of the *C. jejuni* Δ*htrA* mutant and genetically complemented *htrA* strain.** Serial dilutions (10^−1^, 10^−2^, 10^−3^, 10^−4^, 10^−5^, and 10^−6^) of the indicated *C. jejuni* strains [NCTC11168 wild-type (WT), NCTC11168Δ*htrA* and complemented NCTC11168Δ*htrA/htrA*] with an OD_600_ of 0.1 were spotted in 10 μl volumes onto Müller-Hinton agar plates. The plates were incubated for 3 days in jars under microaerobic conditions at **(A)** 42°C, **(B)** 44°C, or **(C)** 42°C in the presence of 18% O_2_. Representative sections of the agar plates from three independent experiments are presented.
