# Supplementary figures and images for: The role of serine protease HtrA in acute ulcerative enterocolitis and extra-intestinal immune responses during Campylobacter jejuni infection of gnotobiotic IL-10 deficient mice
Source: Front Cell Infect Microbiol. 2014 Jun 10;4:77. doi: 10.3389/fcimb.2014.00077 (PMC4050650; doi:10.3389/fcimb.2014.00077)

# Supplemental Figure S1

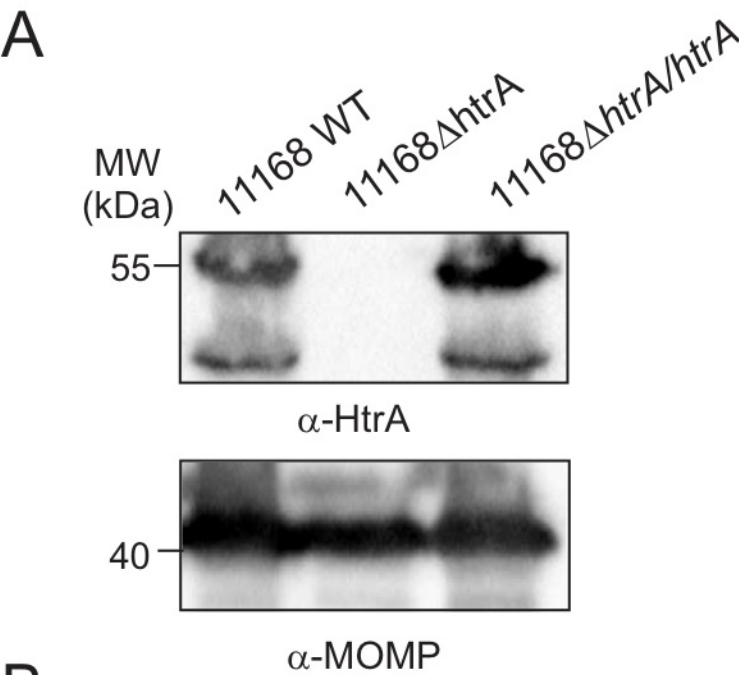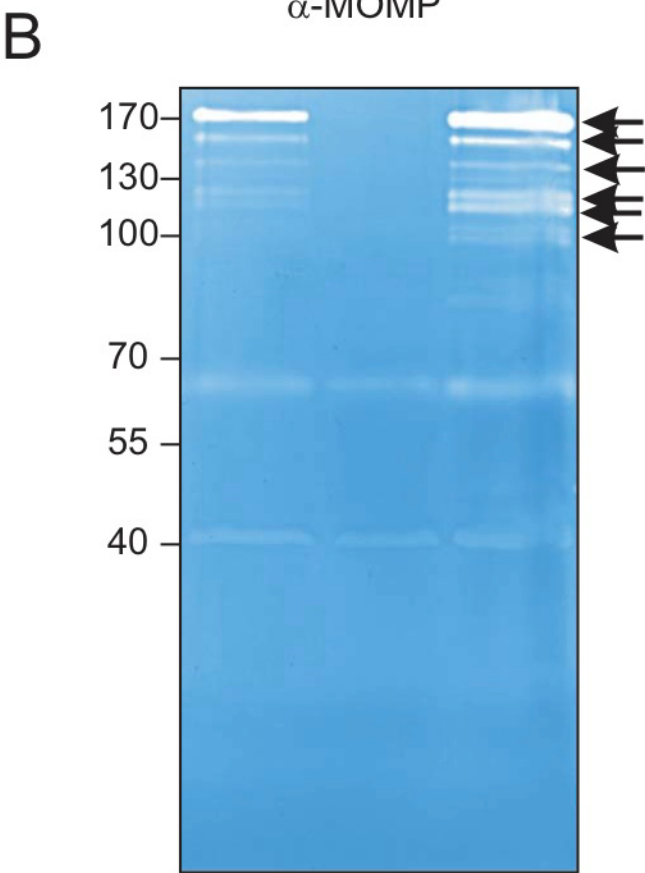

Supplement: Figure S1 — Genetic complementation of HtrA reveals that the C. jejuni NCTC11168 ΔhtrA mutant is non-polar. (A) Expression of HtrA proteins by C. jejuni NCTC11168 wild-type (WT), NCTC11168ΔhtrA mutant and complemented NCTC11168ΔhtrA/htrA were investigated by Western blotting using an α-HtrA antibody. As control, equal amounts of protein per sample were confirmed by immunoblotting using the α-MOMP antibody. (B) Analysis of protease activities in all three indicated strains by casein zymography. The position of proteolytically active multimeric HtrA proteins is indicated with arrows. [file Presentation1.PDF]

# Supplemental Figure S2

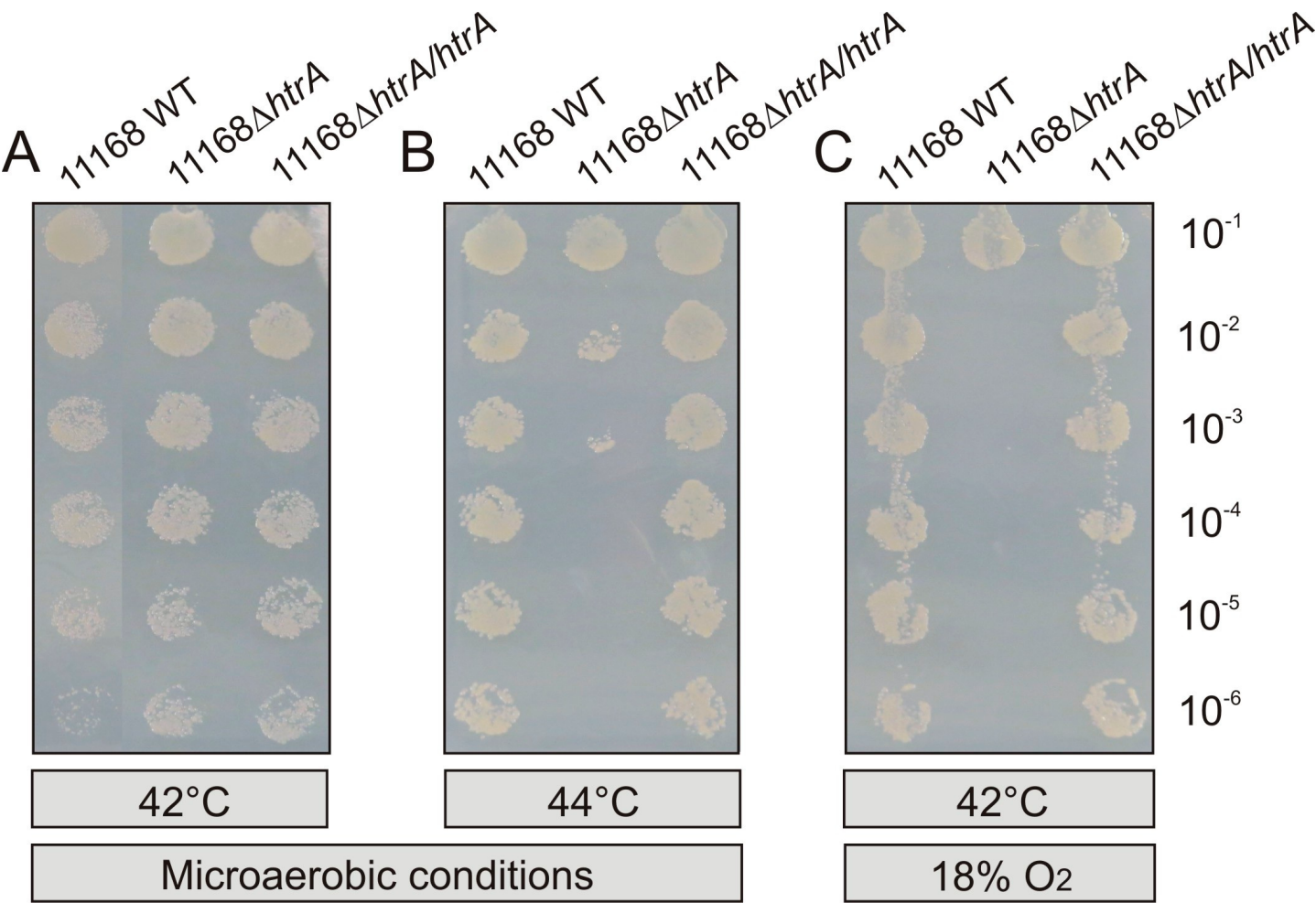

Supplement: Figure S2 — Effect of high temperature and oxygen concentration on growth of the C. jejuni ΔhtrA mutant and genetically complemented htrA strain. Serial dilutions (10−1, 10−2, 10−3, 10−4, 10−5, and 10−6) of the indicated C. jejuni strains [NCTC11168 wild-type (WT), NCTC11168ΔhtrA and complemented NCTC11168ΔhtrA/htrA] with an OD600 of 0.1 were spotted in 10 μl volumes onto Müller-Hinton agar plates. The plates were incubated for 3 days in jars under microaerobic conditions at (A) 42°C, (B) 44°C, or (C) 42°C in the presence of 18% O2. Representative sections of the agar plates from three independent experiments are presented. [file Presentation2.PDF]
